# Fire-adapted Gondwanan Angiosperm floras evolved in the Cretaceous

**DOI:** 10.1186/1471-2148-12-223

**Published:** 2012-11-22

**Authors:** Byron B Lamont, Tianhua He

**Affiliations:** 1 Department of Environment and Agriculture, Curtin University, PO Box U1987, Perth, WA 6845, Australia; 2 School of Environmental Science, Murdoch University, Murdoch, WA 6150, Australia

**Keywords:** Fire, Paleoecology, Adaptive traits, Proteaceae, Seed storage, Cretaceous

## Abstract

**Background:**

Fires have been widespread over the last 250 million years, peaking 60−125 million years ago (Ma), and might therefore have played a key role in the evolution of Angiosperms. Yet it is commonly believed that fireprone communities existed only after the global climate became more arid and seasonal 15 Ma. Recent molecular-based studies point to much earlier origins of fireprone Angiosperm floras in Australia and South Africa (to 60 Ma, Paleocene) but even these were constrained by the ages of the clades examined.

**Results:**

Using a molecular-dated phylogeny for the great Gondwanan family Proteaceae, with a 113-million-year evolutionary history, we show that the ancestors of many of its characteristic sclerophyll genera, such as *Protea*, *Conospermum*, *Leucadendron*, *Petrophile*, *Adenanthos* and *Leucospermum* (all subfamily Proteoideae), occurred in fireprone habitats from 88 Ma (83−94, 95% HPD, Mid-Upper Cretaceous). This coincided with the highest atmospheric oxygen (combustibility) levels experienced over the past 150 million years. Migration from non-fireprone (essentially rainforest-climate-type) environments was accompanied by the evolution of highly speciose clades with a range of seed storage traits and fire-cued seed release or germination mechanisms that was diagnostic for each clade by 71 Ma, though the ant-dispersed lineage (as a soil seed-storage subclade) was delayed until 45 Ma.

**Conclusions:**

Focusing on the widespread 113-million-year-old family Proteaceae, fireproneness among Gondwanan Angiosperm floras can now be traced back almost 90 million years into the fiery Cretaceous. The associated evolution of on-plant (serotiny) and soil seed storage, and later ant dispersal, affirms them as ancient adaptations to fire among flowering plants.

## Background

Recent syntheses have highlighted geological evidence for the occurrence of fire since the Triassic, particularly over the last 65−125 million years (Cretaceous) [1], suggesting that fire may have had a major role in the evolution of Angiosperms [2-4]. However, fire as an important agent of natural selection has received little attention because fireprone floras are usually thought to have appeared only after the global climate became more arid and seasonal <15 million years ago (Ma) making their fire-related traits ‘exaptations’ at best [5,6]. Recent trait-assignment studies have shown that fireprone floras likely existed in Australia [7,8] and the Cape of South Africa [9,10] earlier than that (up to 60 Ma) coinciding with the appearance of fire-adapted traits. However, estimates of the time of origin of ancestral fire-related traits and fireproneness of the habitat occupied by the root of the clade may still have been constrained by the a) insufficient age of the clade, or b) inability of the DNA in extant organisms to adequately reflect the fossil record because of lineage (ancestral DNA) loss. That is, the estimated time of origin of fire-related traits or fireprone habitats may be limited by the estimated age of the clade not necessarily by the inauguration of recurrent fires (though a cause-and-effect coincidence is possible).

**Figure 1:**
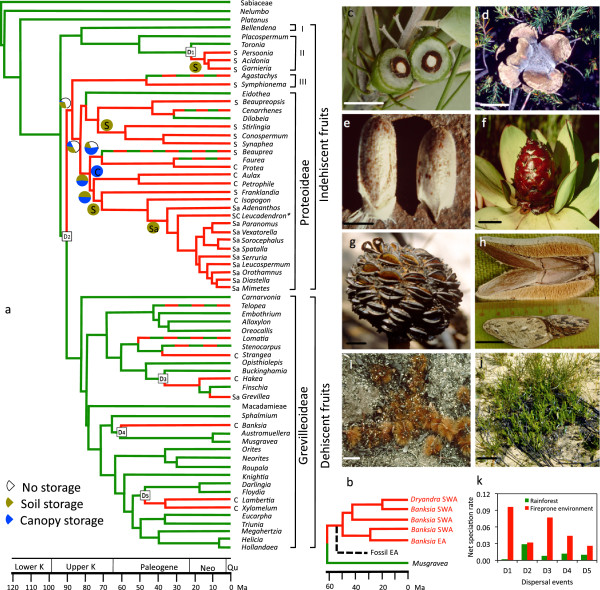
**Chronophylogeny and trait assignment for the Proteaceae.** (**a**) genera and lineages that currently occur in fireprone floras (with probability >0.95) are given in red, with broken lines indicating lineages with both extant rainforest and fireprone-habitat species. Data for phylogeny from [12] with the position of *Banksia sl* updated [8, Figure 1b. Genera in Macadamieae were collapsed into one lineage. I = Bellendenoideae, II = Persoonioideae, III = Symphionematoideae (nomenclature after [49]). K = Cretaceous, Neo = Neogene, Qu = Quaternary. S = soil storage diagnostic for the genus, C = canopy seed storage (serotiny), Sa = soil storage plus diaspore has aril, * S, C and Sa present. Sectors of disks represent fractions of total probabilities of each seed-storage type along that stem. S, C and Sa within the chronogram are the condition of the clade at that position with probability > 0.95. D1−5 are divergence events where the clade splits between rainforest and fireprone habitats with probability > 0.80. (**b**) abbreviated chronogram for *Banksia ss* and *Dryandra* (from [8]) with extinct fossil banksias in Eastern Australia (EA) given as a broken line. (**c**) drupe of *Persoonia saccata* showing ‘stone’ (pyrene) that survives digestion and is promoted to germinate by fire, scale 1 cm. (**d**) cone of *Banksia laricina* with follicles that open in response to heat produced during combustion of its fine foliage, 2 cm. (**e**) diaspore of *Adenanthos cygnorum* showing ant-attracting aril at its base (left) and removed by ants (right) that is buried and stored until stimulated to germinate by fire, 2 mm. (**f**) cone of *Leucadendron tinctum* that releases its winged diaspores on desiccating after fire, 2 cm. (**g**) cone of *Banksia lanata* postfire showing open follicles with (upper left) the separator easing out two winged seeds in response to wet-dry cycles, 2 cm. (**h**) obligately heat-opened follicle of *Xylomelum angustifolium* showing mottled seed that suggests crypsis when among post-fire litter particles, 2 cm. (**i**) serotinous diaspores of *Protea burchellii* released onto an ash/charcoal microsite, 1 cm. (**j**) postfire litter microsite showing 3-month-old seedlings of serotinous *Banksia*, *Hakea* and *Petrophile* species, 10 cm. (**k**) net speciation rates for 5 sister rainforest/fireprone lineages/clades (D1−D5 in Figure 1).

By way of example, trait-assignment analysis of *Dryandra* (family Proteaceae) records fire in southwestern Australia from 20.5 Ma as it originated then but its co-occurring parent group, *Banksia ss*, records fire in southwestern Australia (SWA) much earlier from 61 Ma ( [8], Figure 1b). Equally, fire might have been present in Australia earlier but 61 Ma is the limit of the age of the *Banksia* clade so cannot be used to detect it any earlier. The same constraint applies to *Disa* when inferring age of fire in the Cape [9,10]. In addition, molecular analysis shows that fire-adapted banksias were in Eastern Australia from 25.5 Ma [8], yet pollen and leaf fossils are known from there (and even New Zealand) since at least 56 Ma [11], Figure 1b. This suggests that waves of extinction/migration have occurred in eastern Australia, such dynamics being missed by a simple molecular analysis because the relevant lineages have been lost.

Thus, inferring the time of origin of fireprone floras using individual genera within a family can be artificially constrained by extinction of certain lineages or age of the clade itself, ignoring the possibility that it was a change in the fire regime that stimulated the evolution of the clade in the first place. However, the family Proteaceae can be traced to 113 Ma (Lower Cretaceous) [12] and holds promise as a demonstration of unconstrained evolutionary responses to fire as it is considered to have rainforest-climate (non-fireprone) origins and its extant fire-adapted traits in fireprone floras are well known. Seeds stored on the plant (serotiny) and released via fire heat [13], or transferred to the soil via wind or ants [14] and cued to germinate by fire heat or smoke [15], Figure 1c−i, are critical functional traits in fireprone environments [3,16,17], as they enable seeds to germinate in response to fire when recruitment conditions are optimal (Figure 1j). Hence, we may view the various types of seed storage/release/germination as fire-adapted traits, in the sense of increasing fitness in the presence of fire [16]. Seed storage/germination type is a conserved generic trait so that we were able to superimpose the three major syndromes of seed storage/germination (serotiny; soil – all dispersal types, ant-dispersed only) onto a recently produced Proteaceae chronogram (Figure 1a). Critically from an adaptation perspective (i.e. having evolved in response to the purported agent of selection, 16), we sought to test whether the three types of seed storage have evolved synchronously with the onset of fireprone habitats occupied by this family. If this were so it would be the first evidence of fire-adapted traits occurring among flowering plants as far back as the Cretaceous, a time of high flammability and escalating species diversification rates [1,4].

We chose seed storage/release/germination traits as these are well documented for the entire family and are invariably diagnostic for each genus, and difficult to interpret as other than fire-adapted [8,13]. Other putative fire-related traits that also have been examined in proteaceous genera, such as dead leaf retention, fire-stimulated flowering and resprouting [8,10], have the disadvantages that they are poorly documented by comparison (e.g. rainforest species never burn to determine if they could resprout), are not clearly ancestral within the genus or definitely derived [8,10], and/or not yet convincingly shown to have greatest fitness in the presence of recurrent fire. Much more research at the level of documenting occurrence of these traits and demonstrating superior fitness at the species level is required before their evolutionary history can be considered further.

## Results

Analysing the entire family Proteaceae confirmed that it was able to provide an unconstrained estimate of the time fire-related traits and fireprone floras originated (Figure 1a). Our ancestral state reconstructions, using both range expansion and fireprone trait allocation approaches (see Methods), were the same, showing that the origin of fireprone proteaceous floras can be traced to 88 (83−94, 95% HPD) Ma, at the root of the indehiscent clades (subfamilies Proteoideae and Symphionematoideae) and 25 million years (My) younger than the origin of the family clade [12]. This time coincided with the migration of shrubby, fine-leaved ancestors from closed forest to fireprone habitats (D2 in Figure 1a). Thus, the first 25 My existence of the Proteaceae clade was confined to closed forest, but not until the indehiscent-fruited ancestors of the Proteoideae colonized fireprone sclerophyll woodland/forest (and later scrub- and mallee-heath) did its genera and species proliferate (Figure 1a). This difference in the pattern of speciation between fireprone and non-fireprone habitats was not the same for the dehiscent-fruited clade (Grevilleoideae) at the generic level but it was at the species diversification level within genera. For the entire family we identify five dispersal events from rainforest to fireprone environments that usually led to explosive speciation among their lineages, on average 4.4 times the rate in closed forest (Figure 1k). There appear to be six genera showing migration events in the reverse direction although four retained species or populations in fireprone habitats, two remained monotypic and *Dilobeia* has only three species (Figure 1a).

During the Coniacian−Campanian Stages of the Upper Cretaceous (geological boundaries according to the International Commission on Stratigraphy, see http://www.stratigraphy.org), fireprone clades appear with a wide array of seed storage/ release/germination responses to fire, especially in the Proteoideae. If seed storage as a trait is substituted for soil or canopy storage (as only binary traits may be analysed) then there is a minimum 0.40 probability of some form of storage at 88 Ma to certainty at 76 Ma (Additional File 3: Figure S3). The assignments indicate that the 70−90 Ma period was a time of much initiation, proliferation and extinction of traits among the early clades. By 74 (71−78) Ma, the stem of the *Petrophile*−*Aulax* clade suggests that serotiny may have arisen in the Australian region of Eastern Gondwana [8] while *Banksia* is the oldest extant serotinous genus there at 61 (56−67) Ma. Serotiny appeared last in the archetype sclerophyll genus, *Hakea*, arising independently from rainforest ancestors 16 Ma (Additional File 1: Figure S1). The stem of the Cape Proteeae (excluding *Protea*−*Faurea*) arose from Australian ancestors [12], so it is less clear for how long the Cape flora has been fireprone. The habitat type of the stem of *ProteaFaurea* is fireprone because of the fireprone status of the surrounding lineages (plus *Protea*) but it is non-serotinous (unlike *Protea*) and lacks storage. At 49 (28−67) Ma, *Aulax* is the oldest extant genus in fireprone habitats of the Cape.

The *Franklandia–Mimetes* clade (SWA) originating 74 (71−78) Ma is the oldest with soil storage (Additional File 2: Figure S2). This is followed almost immediately, and independently, by the *Stirlingia*−*Conospermum*−*Synaphea* clade (SWA) originating 71 (60−80) Ma. *Adenanthos*−*Leucadendron* to *Mimetes* is the only clade in the Proteoideae with an aril (elaiosome) attached to the single-seeded fruit as an aid to dispersal and burial by ants (Figure 1e, S3). The stem of this elaborate dispersal/storage/germination response to fire is dated from 44.5 (32−58) Ma for *Adenanthos* in SWA and the *Leucadendron* to *Mimetes* subclade in the Cape (as the oldest member of this group in the Cape, *Leucadendron* actually has a wide array of storage mechanisms). These are among some of the earliest elaiosome-bearing lineages known and correspond to the advent of ants as reliable dispersal agents [14]. In contrast, the highly speciose genus, *Grevillea*, was the only Grevilleoid to develop ant dispersal traits and this appeared very late (11 Ma). Overall, each of the three seed storage syndromes evolved closely and synchronously with fireprone habitats in this family (*χ*^2^ = 47.8 (serotiny), 55.4 (soil seedbank), 79.5 (ant-dispersed), *P* < 0.001, [18]).

## Discussion

**Figure 2:**
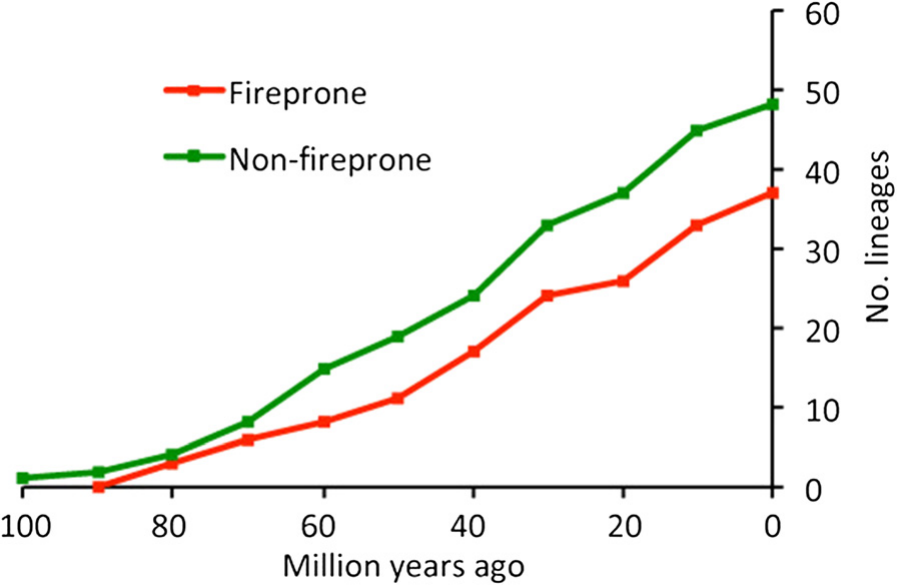
**Proliferation of lineages assigned to fireprone and non-fireprone habitats at 10 My intervals from the estimated time of origin of the Proteaceae (collated from Figure**1a**).**

Flowering plants had small beginnings in Gondwana during the Mid-Cretaceous (100 Ma) but the Proteaceae was abundant in Antarctic temperate rainforests by the Campanian (71−84 Ma) [19,20]. However, our analysis shows that recurrent fire must already have been influencing the path of evolution among Proteaceae by the Mid-Upper Cretaceous (84−89 Ma) and has continued as a strong agent of selection ever since, based on the continual appearance of fire-tolerant clades (Figure 2). The chronogram and trait assignment analysis indicate that ancestors to the dry indehiscent-fruited section of the family, the subfamilies Proteoideae and Symphionematoideae, were already present in fireprone habitats by that time.

Did these clades actually exist by then? The fossil record supports the existence of pollen with affinities to at least two extant genera in the Proteoideae, and 17 pollen types whose Proteaceae affinities are less prescribed, in eastern Gondwana (Zealandia, southern Australia – especially the Otway Basin,) during the Santonian-Campanian (86−71 Ma) [12,19-24]. Dettmann and Jarzen [22] concluded that the southern Australian/Antarctic vegetation in this period must have been a mosaic of rainforest (though the angiosperm element would not have been dominant) and sclerophyll communities, with the Proteaceae prominent in the latter (Sauquet et al. [12] discredit most of their identifications at genus level but this does not invalidate their clear Proteaceae affinities that are not in dispute).

Did suitable habitats exist by then? By 90−85 Ma most of the current Australian portion of Eastern Gondwana had emerged due to sedimentation (high rainfall) and general elevation of the continent [25], including the Otway, Eromanga and Murray Basins. The sedimentary basins: Eucla, Officer and Surat, were already exposed by 100−90 Ma. In addition, various metasediments were uplifted/downwarped in the lower-mid-Cretaceous (Stirling−Barren Ranges in SW Australia, Great Dividing Range, Victorian Southern Uplands [6,26]). Heavy leaching due to the high but seasonal rainfall during the Upper Cretaceous created oligotrophic sands and ferricretes [22,27,28]. There has even been the suggestion that the Proteaceae was a (mainly Cenozoic) driver in this process [29]. Thus, well-drained, nutrient-poor substrates (sands, sandstones, quartzite, schists, laterites) of variable elevation and suitable for colonization by the evolving sclerophyll element were already widespread.

**Figure 3:**
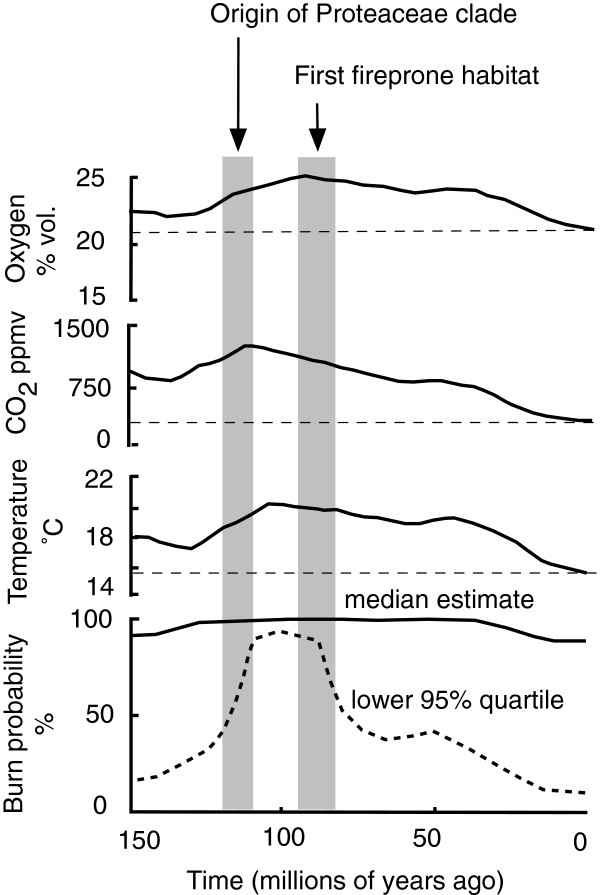
**Mean atmospheric conditions over the last 150 million years.** From top to bottom, oxygen and carbon dioxide concentrations, annual global temperature, and ignition probability of plant matter. Broken horizontal lines correspond to ambient levels. Shaded bands correspond to the origin of the Proteaceae clade and first fireprone habitat (with 95% HPD bounds) from [12] and Figure 1a. Adapted from [1].

Were these habitats also fireprone? Not only were carbon dioxide levels and mean temperatures much higher than currently (favouring high productivity) [30] but calculated oxygen levels 80−90 Ma were 25% higher (Figure 3, [4]), all greatly enhancing flammability. Bond and Scott [3] note in addition strong seasonality, frequent lightning and charcoalified fossils peaking at 65–105 Ma, though these values are biased towards the Northern Hemisphere. The presence of indehiscent-fruited Proteaceae in fireprone habitats for the first time at 88 Ma coincides with the peak or just postpeak atmospheric levels of oxygen, carbon dioxide, temperature and burn probability in the presence of an ignition source over the last 150 My (Figure 3, [31]), bearing in mind that crown Angiosperms appeared about 135 Ma [32]. The nutrient-impoverished substrates where sclerophyllous Proteaceae are favoured would have supported dense, shrubby evergreen vegetation with highly flammable foliage particularly vulnerable to lightning ignition at this time. The Proteoideae itself would have contributed to this flammability: small, acicular or highly divided isolateral leaves, finely divided foliage, highly sclerophyllous, long-lived leaves with low nutrient content and poorly decomposable litter [33,34].

Is there direct evidence of fire at that time? Abundant charcoal is recorded in the Eromanga Basin, SW Queensland, at 100–94 Ma [35]. Charcoal was noted among pollen samples in Zealandia at 94–85 Ma [36], together with other evidence “suggesting a generally broad occurrence of fire in the mid-Cretaceous across middle to high latitudes in the Southern Hemisphere” that the authors attributed in part to the high atmospheric oxygen. Charcoalified fragments (‘black wood’) made up 40% of the microsamples that contained pollen of > 7 Proteaceae species (including several with suggested Proteoideae affinities) in Zealandian sandstones at 88–71 Ma [24] indicating that “wildfires were part of the Late Cretaceous ecosystem”. Eklund et al. [37] studied 83-My-old charcoalified mesofossils of Angiosperms between Antarctica and Australia that they attributed to fires caused by volcanism near the collection site.

Was there selection against the fire-sensitive ancestors that might have biased the assignments? Figure 2 shows that net lineage proliferation at the generic level has continued steadily and in parallel for both fireprone and non-fireprone groups. This means that fire-sensitive clades did not become extinct at the expense of fire-tolerant clades that might have distorted the analysis – this is consistent with both being accommodated in different vegetation types through time preventing their extinction [23]. If extinction was *recent* it would have to be on a massive scale to delete all trace of the non-fire-tolerant lineage. It seems that even during the last glacial maximum, rainforest species in eastern Australia, including Proteaceae, were able to survive in moister lowland refugia [18]. Further, if (unrecorded) extinction of fire-sensitive taxa had occurred along *basal* stems of clades that our analysis currently assigns as in fireprone habitats, this would not alter the assignment as this (correctly) infers that the surviving lineage was fire-tolerant at that point in time. The only requirement then is to show that the lineage actually existed at that time via fossil evidence. In this regard it is noteworthy that 15 of the 26 pollen/leaf types assessed by Sauquet et al. [12] were allocated to the base of extant rainforest clades or sediments arising > 88 Ma, i.e. prior to the oldest time that our analysis identifies fireprone habitats for the Proteaceae. In addition, five are associated with younger rainforest clades, ten with fireprone habitats, and all 26 can be traced to extant genera (though few could be confidently assigned to any one), i.e. none necessarily implies an extinct lineage.

A related problem with the purported origin of the three extant fire-tolerant Proteaceae lineages in the Upper Cretaceous (Figure 1a) is the possible exterminatory effect of the subsequent early Eocene (40–56 Ma), one of the wettest periods known. Indeed, fire-derived inertinite in coal dropped markedly in Northern Hemisphere mires after 60 Ma [3]. While the climate was especially wet and aseasonal throughout eastern Gondwana during the Eocene, it remained warm (except in the Tasmanian region) and much of the Central-West zones of the Australian portion of Gondwana had moderate and strongly seasonal rainfall [39]. Atmospheric oxygen levels were up to 15% higher than currently and burn probability was the highest for the last 60 My (Figure 3). Further, deeply weathered sands or siliceous/lateritic rocks, often in open, well-drained parts of the landscape, were widespread and would have provided ideal refugia for the sclerophylls, especially Proteaceae, in their own communities [38,40-42]. Indeed, the Proteaceae (*Banksia*-like wood, pollen akin to *Petrophile*, *Adenanthos*) at Lake Lefroy, SWA in the mid-Eocene accounted for 34% of all pollen (when *Nothogfagus* was omitted), with species richness values comparable with those in SWA scrub-heath today [42]. Interestingly, where fire did occur around the eastern Otway Basin, it not only led to a severe reduction in *Nothofagus* but also in Proteaceae and Myrtaceae [43], implying that the species there were more vulnerable to fire than their western counterparts [42].

We therefore suggest that much of the fire-dependent, sclerophyll element contracted west during the Eocene. Some of these returned/expanded in a second wave of migration during the Oligocene-Miocene (e.g. *Banksia* ss, *Eucalyptus*) but usually remained poorly represented (e.g. *Adenanthos*, *Petrophile*), others did not return (e.g. *Franklandia*), while others only arose then but never reached eastern Australia (e.g. *Dryandra*). In support, Figure 3 shows no increase in proliferation rates of non-fireprone-habitat lineages at the expense of fireprone lineages in the 40–60 My period; if anything the reverse is true. In addition, we point to the exacerbated bias against increasingly isolated pockets of sclerophyll vegetation in the fossil record from lake sediments during particularly wet periods, and note that strongly fire-adapted *Pinus* in the NH (fossils) and *Nuytsia* in SWA (molecular dating) have also been recorded from the mid-Eocene [1,10].

Finally, the two seed storage mechanisms that facilitate postfire germination and seedling recruitment were already appearing 88−81 Ma according to our analysis, though neither type prevailed in any clade, indicating that much diversification and extinction along the major stems must have been occurring. By 81 Ma, seed storage was firmly established in one basal clade. By 71 Ma, clades in fire-prone habitats were characterized by either soil or on-plant fruit storage (except for continuing variation in the *Beauprea*/*Faurea*/*Protea* clade). Note that the two rainforest genera in Proteoideae, *Eidotheia* and *Delobeia*, did not speciate to any extent (or suffered almost complete extinction), while two remained monotypic (*Agastachys*, *Cenarrhenes*) or had limited speciation (*Beauprea*, *Faurea*) in genera with populations or species respectively largely remaining in rainforest today. In particular, only *Beauprea* among the seven genera occupying mixed habitats has the capacity for seed storage, though it would be of interest to know why this once-widespread genus became extinct in Australia. The only genera lacking strong speciation once in fireprone habitats are *Beaupreopsis*, with a small soil-stored pyrene, that occurs in scrub-heath of New Caledonia and is monotypic (44), and *Symphionema* with two species in scrub-heath of NSW and possesses a thin-walled achene “expected to show some form of dormancy” [45]. Comparing speciation rates overall between fireprone-habitat and non-fireprone sister clades (Figure 1k), the pivotal role of seed storage for successful occupation of fireprone habitats is supported (Figure 1a) and the two are highly correlated through time (*P* < 0.001) confirming their classification as true fire adaptations.

The mechanism of seed storage on the plant is different between the Grevilleoideae and Proteoideae: in the former, woody fruits protect and support the seeds and dehisce in response to heat and desiccation while, in the latter, bracts (assisted by branchlets in the case of *Aulax*) protect and support the fruits and spread to release the single-seeded fruits in response to heat and desiccation. This has arisen four times independently in the Grevilleoideae, and four times in the Proteoideae (Figure 1a). While they were treated as the one trait for purposes of the analysis this did not create origin artefacts as the two subfamilies remained independent with respect to the evolution of serotiny in the family.

All soil-stored seeds are actually one-seeded fruits in the Proteaceae (except *Grevillea*), comprising a thin pericarp (swollen at the base to form an elaiosome in the Adenanthos–Mimetes clade, Figure 1a), exotesta (of varying thickness and texture) and crystalliferous endotesta [46,47]. The succulent-fruited Persoonieae (Persoonioideae) had an independent origin from the dry-fruited Proteoideae/Symphionematoideae that appeared independently five times (Figure 1a). *Leucadendron* has a possible sixth independent origin where our informal analysis of a cladogram for 62 of its 96 taxa [48] indicates that the ancestral state is serotiny with winged fruits followed by dormant nutlets and soil-stored achenes, followed much later by a few species with elaiosome-bearing achenes – this requires more detailed research, especially the need for smoke-stimulated germination throughout the genus. This is the only genus that had to be assigned mixed traits – in all others the assigned trait was part of the circumscription of the genus or shown to be the ancestral condition (*Banksia*[8]; *Protea*, Lamont and He unpublished). The separate origins of soil-storage in the *Stirlingia–Synaphea* (71 Ma) and *Franklandia–Mimetes* (74 Ma) clades do not merge in the analysis (Figure 1a). *Grevillea*, with its exotesta modified into a brittle wing or aril attractive to ants, is unique among the dehiscent Proteaceae and had no effect on the outcomes for ancestral assignments of soil-storage.

## Conclusions

Fireproneness among Gondwanan floras can now be traced back to the fiery Cretaceous and trait assignment affirms serotiny and soil seed storage, and later ant dispersal, as ancient adaptations to fire among flowering plants. Most species in over 90 extant flowering-plant families of the sclerophyll vegetation in Australia have some form of seed storage, and up to 25,000 seeds from many families are stored per m^2^ of soil in fireprone sclerophyll communities throughout the world [49]. This means that other clades and floras should now be examined to see if they have a similarly ancient relationship with fire as a vital agent of natural selection favouring seed storage. Since Proteaceae is among the oldest of the eudicot families and only monocots (five orders have fire-tolerant members) migrated into fireprone habitats among older clades [10], it will be interesting to see if dates older than 88 Ma for fire-related traits can be identified among flowering plants. He et al. [1] have shown recently that fire-related traits appeared among pines (non-flowering) up to 126 Ma, but especially from 89 Ma when canopy seed storage originated in association with more intense, crown fires. The similarity of these two dates (c. 90 Ma), with one family confined to the Northern Hemisphere and the other to the Southern, is remarkable. However, other flowering plant orders/families well known for their fire-tolerant traits also arose around this time (see [1] for details) and this raises the prospect that we may have identified a time of great significance in the fire history of seed plants generally.

## Methods

### Dataset

Sauquet *et al.*[12] constructed a dated phylogeny for the 81 currently recognised genera in the Proteaceae. The age of each lineage was determined by Bayesian autocorrelation dating based on eight DNA regions and five fossil pollen age calibration points. We used the tree they obtained by the uncorrelated lognormal method (implemented in BEAST), as this was preferred by them since it produced similar age estimates to the other two methods they tried but had the advantage that it took phylogenetic uncertainty into account. The calibration points were determined after analysing 25 fossil palynomorph species, and a morphological matrix of 22 pollen characters and 113 taxa, to produce a highly accurate dated phylogeny for the family. The crown age of Proteaceae was dated at 113 Ma (108−118 Ma, 95% highest posterior density). The maximum clade credibility tree (with 86% of internal nodes at generic level having a posterior probability greater than 0.95) was used for downstream analysis.

The presence of fire-related seed storage/germination traits varies greatly in the Proteaceae. The key to maximum fitness is seed storage/release/germination cued to fire and establishment immediately after fire when the recruitment conditions are optimal. There is a wide array of seed storage/germination responses to fire, especially in Australia: ‘stones’ (pyrenes) that lie near the soil surface until stimulated to germinate by heat [50]—*Beauprea* (may occur in fireprone maquis of New Caledonia [44] but was once widespread in Australia), Persoonieae (Figure 1); woody follicles and ‘cones’ that release their buoyant, nutrient-enriched diaspores following flame heat [13,17]—Grevilleoideae, Proteoideae; plumed, hairy, leathery or chitinous diaspores that work their way into the soil, or are buried by ants, and germinate in response to smoke or desiccation caused by high soil temperatures during or after fire [15,51]—Proteoideae.

Data on fire-related seed storage/germination for each genus were collated from the literature [13,17,52,53] and our own fieldwork, and categorised as present or absent. If all known extant species within a genus shared a particular fire-related seed storage/germination syndrome then this trait was treated as diagnostic for the genus. Species current distribution in each genus (and outgroups) was compiled from the literature [12,52], and categorised into fireprone and non-fireprone (essentially rainforest) environments that are the two major habitats of extant species in the Proteaceae. A fireprone environment was defined as subject to an average fire interval < 40 years (the mean lifespan of many fire-killed Proteaceae). The only uncertain genus was *Bellendena* that occurs in alpine scrub-heath of Tasmania, which appears subject to occasional fire but probably at > 40 year intervals, so it was assigned to the non-fireprone habitat type. If all known extant species share the same habitat type, this was treated as diagnostic for the genus. Where the habitat is variable within or between species the genus was assigned both traits. Seed and habitat traits for individual genera are provided in Figure 1a.

### Ancestral habitat reconstruction and paleodispersal

Evolution of habitat and paleodispersal events was inferred on a phylogenetic tree of the Proteaceae by a maximum likelihood procedure. Maximum likelihood inference of ancient occupied habitat uses a dispersal-extinction-cladogenesis (DEC) model for habitat evolution that specifies instantaneous transition rates between discrete states (habitat type) along phylogenetic branches and applies it to estimating likelihoods of ancestral states (habitat inheritance scenarios) at the cladogenesis event [54]. Ancestral habitat reconstruction and palaeodispersal was implemented in *Lagrange*. Genera in the Proteaceae were assigned to one of two habitats, non-fireprone (essentially rainforest) and fireprone environments, or both (where different species or populations occupied both habitat types). Dispersal between habitats was set as unconstrained. As an alternative approach, habitat was treated as a generic (heritable) trait as for the seed syndromes and analysed in the same way.

### Ancestral state reconstruction for fire-related seed storage/germination

The tree file containing the maximum credibility clade tree with branch length (node age) generated by the molecular dating procedure, and trait files containing the discrete state for each genus were used for the analyses. Each of the three traits was analysed separately. Posterior distributions of log-likelihoods and the values of traits at the nodes of phylogenies were derived from a Bayesian MCMC method [55]. The continuous-time Markov character model assumes trait states can evolve repeatedly between their possible states at any branch of the phylogenetic tree [55,56] and constructs the ancestral trait at each internal node. This model allows the trait to change from the state it is in at any given moment to any other state over infinitesimally small intervals of time. The rate parameters of the model estimate these transition rates. As recommended, a reversible-jump (RJ-)MCMC was adopted [18]. In RJ-MCMC, the Markov chain searches the posterior distribution of different models of evolution, and the posterior distributions of the parameters of these models.

In the MCMC mode, implemented in *BayesMultiStates*, a new set of rate parameters for the evolution model is proposed at each iteration of the Markov chain by changing the current values by an amount given by the *ratedev* parameter representing the deviation from the normal distribution [18]. Three Markov chains were run for the trait and the acceptance rate was monitored for the newly proposed values of the rate parameters to determine a suitable value for the *ratedev* parameter. A value of *ratedev* was accepted if it generated an acceptance rate of 20−40%. A *hyperprior* seeding an exponential distribution from a uniform 0−30 distribution was also applied in Markov chain runs. The Markov chains were run for 10^7^ iterations with a burn-in of 10^4^, and the result was recorded every 2000th iteration. The ancestral state was defined unambiguously as the trait with the phylogenetic corrected posterior probability from [12] (posterior probability of trait value on the internal nodes × posterior probability of the existence of that node in phylogeny) greater than 0.95.

As this is an agglomerative nonexclusive technique (the opposite of synapomorphy assignment in traditional cladistics), the probability of a given trait assigned to a node is retained along its supporting stem until an assignment decision is made at the next node below it [18,57,58]. The new probability is affected by the length of the stem as well as the surrounding node probabilities, but, in the absence of any further data on the lineage, the trait probability is assumed to remain stable between nodes. This interpretation is consistent with that of other recent studies on clade, trait and paleodispersal assignment [12,57-59] and has not led to any conflict with the fossil record [8,57].

### Evolutionary correlation of fire-related seed storage/germination and fireprone environments

We consider that extrapolating environmental traits into the past is valid only if it is tied to an associated limiting functional trait. We first test whether fire-related seed storage/germination in Proteaceae is positively correlated with fireprone environments over evolutionary time. Evolutionary correlation of fire-related seed storage/germination and fireprone environments was analyzed in *BayesDiscrete*[56]. *BayesDiscrete* tests for correlated evolution between two binary traits by comparing the fit (log-likelihood) of two continuous-time Markov models. One is a model in which the two traits evolve independently on the tree (*independent* model). The other model allows the traits to evolve in a correlated fashion (*dependent* model). We adopted a RJ-MCMC following Pagel & Meade [18]. A few test runs were executed to choose a *ratedev* value that produced an acceptance rate of 20−40% as recommended. Using the chosen *ratedev* value, a reversible jump *dependent* model was first tested. The analysis was repeated by confining the RJ chain to the *independent* model. In each model run, 5 × 10^6^ iterations were implemented and the results were sampled every 2000 iterations. The harmonic mean was used to summarize the overall result. The log-Bayes factor, which is twice the difference between the two harmonic means derived from the *dependent* and *independent* models of evolution, was calculated. The log-Bayes factor is nominally distributed as a *χ*^2^ with degrees of freedom equal to the difference in the number of parameters between the two models [18]. A difference between these two harmonic means greater than 9.49 indicates strong support (*P* < 0.05) for correlation between the two traits [18].

### Net speciation rate

Differentiation in species diversification rate after each palaeo-dispersal was obtained by comparing the net speciation rate in two lineages after the divergence events. The number of species in each genus of Proteaceae was compiled from Sauquet *et al.*[12]. Stem group net speciation rates were calculated as R_st_ = log(N)/t, where N is the number of extant species in the lineage, and t is the stem age [60].

## Competing interests

We declare no competing interests.

## Authors' contributions

BL conceived this project, wrote the bulk of the manuscript and took the photos for Figure 1 and frontispiece. TH contributed to the conceptual development of the work, undertook all analyses, prepared the figures and wrote the Methods section. Both authors have read and approved the final manuscript.

## Supplementary Material

Additional file 1: Figure S1 Proteaceae chronogram with genera and clades assigned to on-plant seed storage (serotiny) highlighted in red. Click here for file

Additional file 2: Figure S2 Proteaceae chronogram with genera and clades assigned to soil seed storage highlighted in red. Click here for file

Additional file 3: Figure S3 Proteaceae chronogram with genera and clades assigned to soil stored seeds with ant dispersal highlighted in red. Click here for file
