# Supplementary figures and images for: Fire-adapted Gondwanan Angiosperm floras evolved in the Cretaceous
Source: BMC Evol Biol. 2012 Nov 22;12:223. doi: 10.1186/1471-2148-12-223 (PMC3534566; doi:10.1186/1471-2148-12-223)

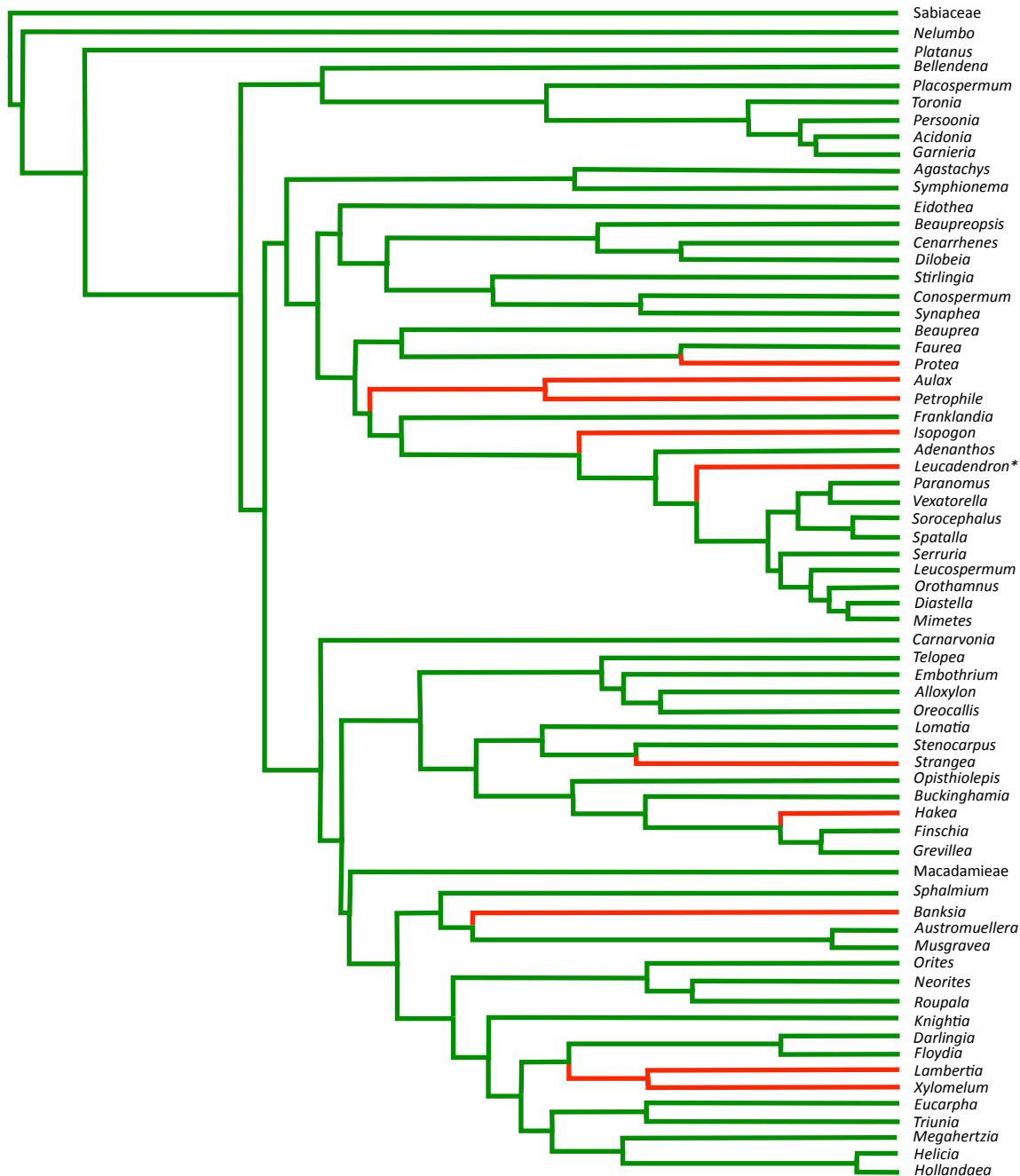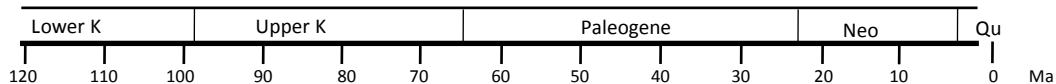

Supplement: Additional file 1: Figure S1 — Proteaceae chronogram with genera and clades assigned to on-plant seed storage (serotiny) highlighted in red. [file 1471-2148-12-223-S1.pdf]

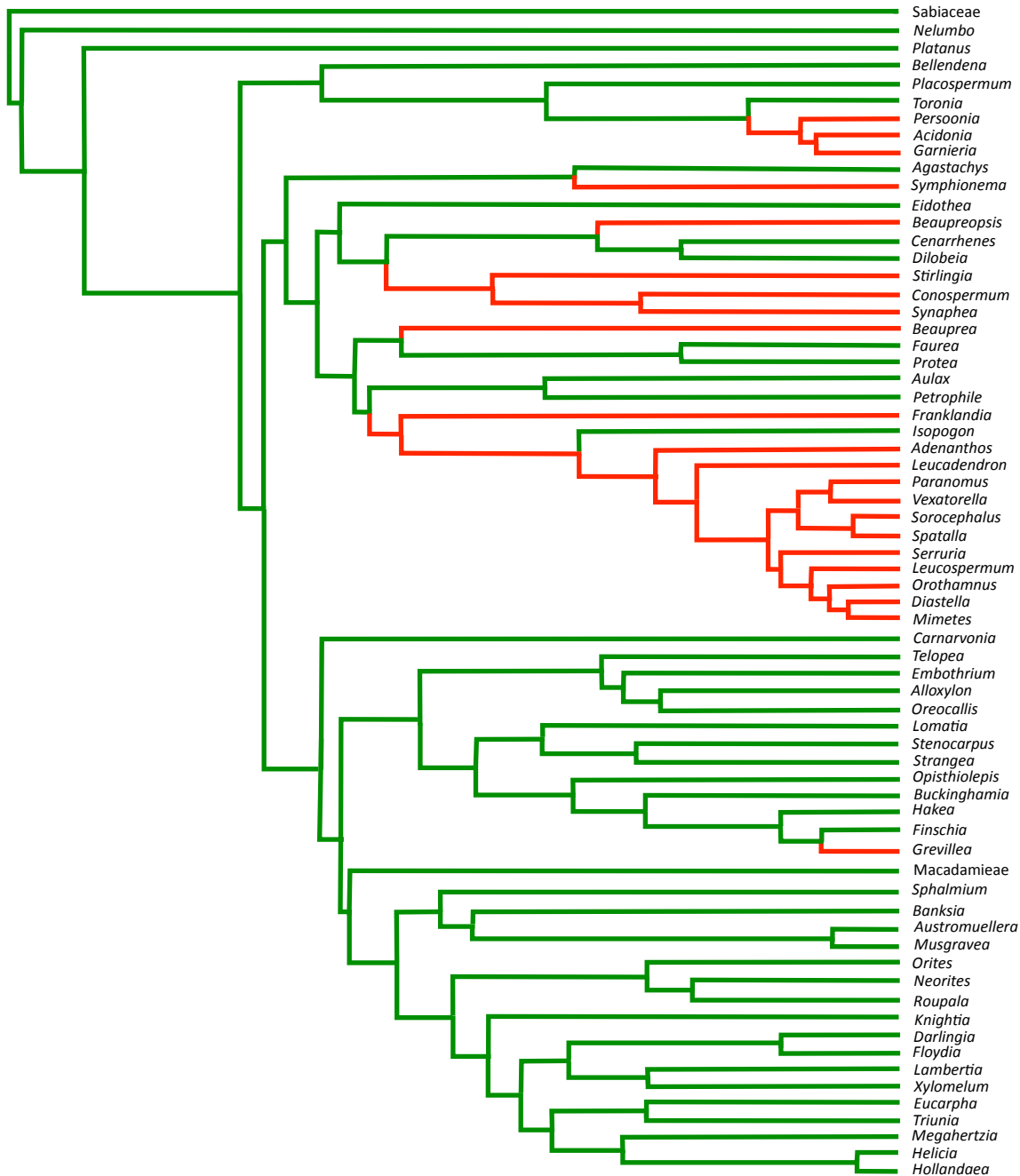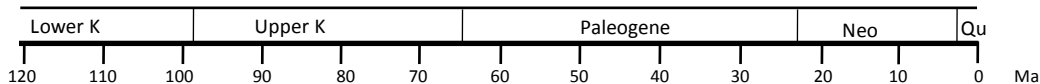

Supplement: Additional file 2: Figure S2 — Proteaceae chronogram with genera and clades assigned to soil seed storage highlighted in red. [file 1471-2148-12-223-S2.pdf]

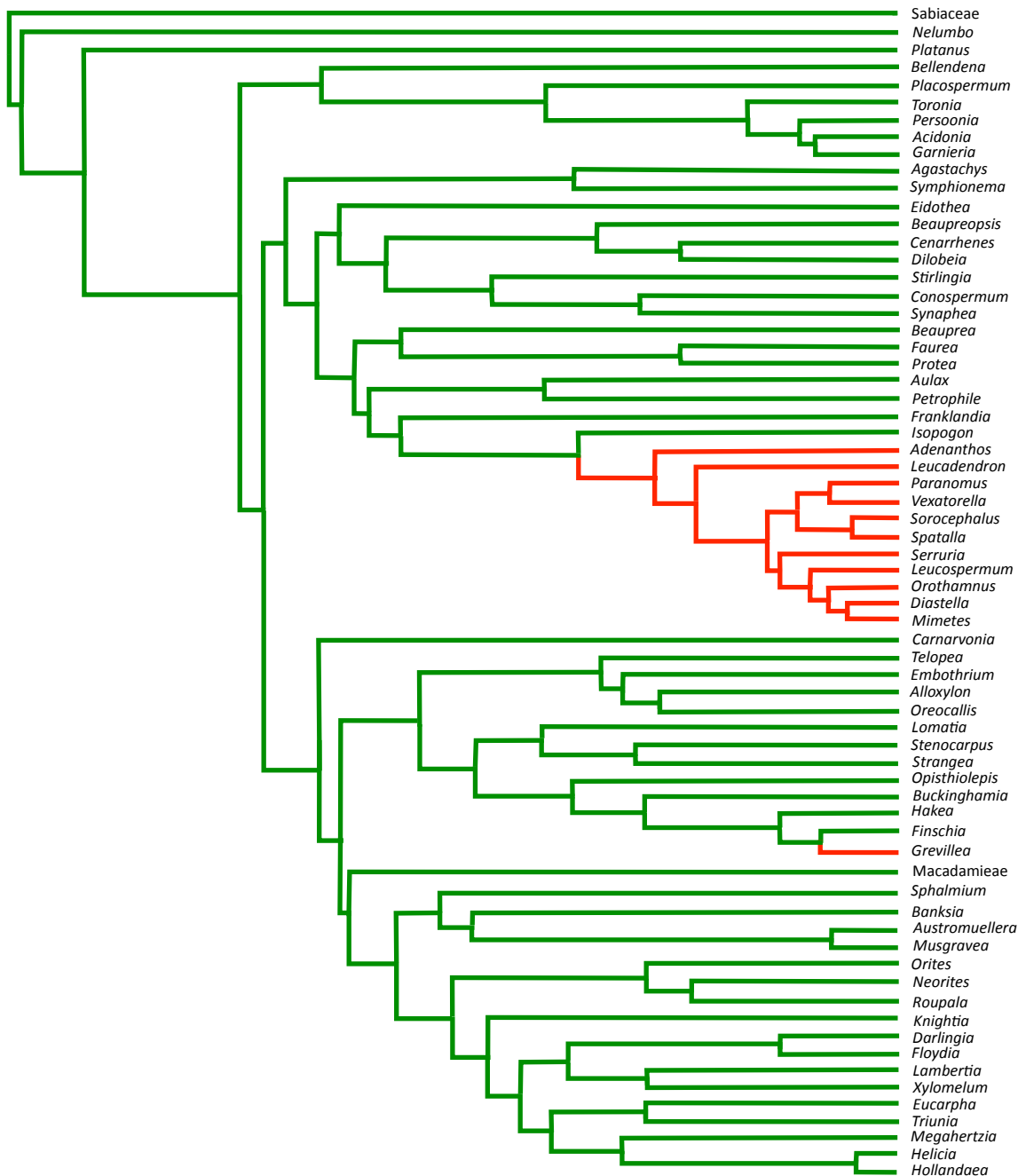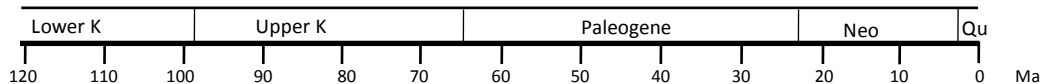

Supplement: Additional file 3: Figure S3 — Proteaceae chronogram with genera and clades assigned to soil stored seeds with ant dispersal highlighted in red. [file 1471-2148-12-223-S3.pdf]
